# On the Sensitivity of the Ni-rich Layered Cathode Materials for Li-ion Batteries to the Different Calcination Conditions

**DOI:** 10.3390/nano10102018

**Published:** 2020-10-13

**Authors:** Hubert Ronduda, Magdalena Zybert, Anna Szczęsna-Chrzan, Tomasz Trzeciak, Andrzej Ostrowski, Damian Szymański, Władysław Wieczorek, Wioletta Raróg-Pilecka, Marek Marcinek

**Affiliations:** 1 Faculty of Chemistry, Warsaw University of Technology, Noakowskiego 3, 00-664 Warsaw, Poland; magdalena.zybert@pw.edu.pl (M.Z.); anna.szczesna@vp.pl (A.S.-C.); tomek.trzeciak@gmail.com (T.T.); aostrowski@ch.pw.edu.pl (A.O.); wladek@ch.pw.edu.pl (W.W.); wiola@ch.pw.edu.pl (W.R.-P.); marekmar@ch.pw.edu.pl (M.M.); 2 Centre for Advanced Materials and Technology CEZAMAT, Warsaw University of Technology, Poleczki 19, 02-822 Warsaw, Poland; 3 Institute of Low Temperature and Structure Research, Polish Academy of Sciences, Okólna 2, 50-950 Wrocław, Poland; d.szymanski@intibs.pl

**Keywords:** Li-ion battery, cathode, Ni-rich layered oxide, NMC, LNO, calcination, heat-treatment

## Abstract

Ni-rich layered oxides, i.e., LiNi_0.6_Mn_0.2_Co_0.2_O_2_ (NMC622) and LiNiO_2_ (LNO), were prepared using the two-step calcination procedure. The samples obtained at different calcination temperatures (750–950 °C for the NMC622 and 650–850 °C for the LNO cathode materials) were characterized using nitrogen physisorption, PXRD, SEM and DLS methods. The correlation of the calcination temperature, structural properties and electrochemical performance of the studied Ni-rich layered cathode materials was thoroughly investigated and discussed. It was determined that the optimal calcination temperature is dependent on the chemical composition of the cathode materials. With increasing nickel content, the optimal calcination temperature shifts towards lower temperatures. The NMC-900 calcined at 900 °C and the LNO-700 calcined at 700 °C showed the most favorable electrochemical performances. Despite their well-ordered structure, the materials calcined at higher temperatures were characterized by a stronger sintering effect, adverse particle growth, and higher Ni^2+^/Li^+^ cation mixing, thus deteriorating their electrochemical properties. The importance of a careful selection of the heat treatment (calcination) temperature for each individual cathode material was emphasized.

## 1. Introduction

Battery technology is significant for the modern world and impacts many aspects of our lives. At almost every step, we use multiple devices, primarily consumer electronics, that require batteries. The Nobel Prize was awarded in 2019 to the creators of this technology—J.B. Goodenough, M.S. Whittingham and A. Yoshino—thus confirming its great importance for the developments necessary for the proper functioning of people in the world and the continuous improvements in the standards of human living. The battery market has developed substantially over the past few decades, and one of the great challenges of the present time is electromobility. This implies a significant increase in the electric vehicle market segment, and thus affects a substantial increase in the demand for lithium-ion battery systems. However, in order to obtain highly efficient batteries, it is necessary to gain a deep understanding of their structure, as well as the phenomena that occur during the operation of these complex, multi-component systems [1,2,3,4,5,6,7,8,9,10,11,12,13].

Nowadays, increasing efforts are being directed towards enhancing the performance of Li-ion batteries. Progress in this area is possible by improving the cathode materials for specific applications. Recently, a noticeable decrease has been observed in interest in the LCO cathode material (lithium cobalt oxide). However, despite its favorable electrochemical properties, the range of applications is limited mostly to small portable devices, e.g., smartphones, tablets, laptops. Electric vehicles or large energy storage systems require batteries with diverse performance parameters, and thus a different composition, mainly with respect to the cathode material responsible for the capacity of the entire cell. Currently, cathode materials with high nickel content [8,9,14,15,16,17,18,19,20,21,22,23] are becoming more and more popular, which is particularly important from an economic point of view; cobalt (the basic component of the LCO material) is an expensive metal and its deposits are limited and hard to access. This group of cathode powders includes LiNi_x_Mn_y_Co_z_O_2_ (NMC) and LiNi_x_Co_y_Al_z_O_2_ (NCA) materials with high nickel content (x ≥ 0.6), formed by partly substituting cobalt with other metals (nickel, manganese, aluminum), as well as LNO (lithium nickel oxide) material—an analogue of LCO with very high theoretical capacity [19,24,25,26,27,28,29,30]. Somehow “abandoned” in the initial phase of the studies of Li-ion batteries because of its serious drawbacks (structural and thermal instability mainly), today LNO is again attracting increasing attention.

The majority of current work on cathode materials is focused on the development of efficient cathode powders with high nickel content, which is their great advantage, while at the same time it has become their biggest disadvantage, and a source of numerous serious problems. Among these, the presence of residual lithium compounds, Ni^2+^/Li^+^ cation mixing (disordering), irreversible phase transition, microcracking of secondary particles or transition metal ion dissolution [20,21] should be mentioned, among others. All of these may cause a deterioration of the electrochemical performance of cathode powders. Nevertheless, the significant benefits in terms of battery capacity that are achievable using these types of high-nickel material are the main reason for the continuous attempts to synthesize them and to improve their properties, e.g., by doping or surface coating [17,31,32,33,34,35,36]. There are many known methods for obtaining cathode materials, e.g., solid state reaction [37], co-precipitation [38], wet-impregnation [39,40], combustion [41], and spray-drying [42]. However, regardless of the chemical composition or the chosen synthesis procedure, the most important stage in the preparation of the cathode powder is its high-temperature heat treatment (calcination) [43,44,45,46,47,48,49]. The parameters of this process, i.e., atmosphere, temperature, time, calcination mode (single-step or multi-step) have a significant impact on the key cathode powders properties such as phase composition, uniform mixing of Li/transition metal ions, purity, particle size, structural defects, and crystal structure [50,51]. This last factor, i.e., a well-developed structure, is particularly crucial for the process of effective intercalation/deintercalation of lithium ions during battery charge/discharge cycles, and is decisive for the capacity of the cathode powder and the efficiency of the cell’s function. For this reason, various aspects of the synthesis process and the heat-treatment of these types of material are still the subject of extensive studies. Literature reports indicate that the selection of appropriate heat-treatment conditions for specific materials determines their structural properties, and thereby their subsequent electrochemical performance [43,44,45,46,47,48,49]. The selection of a calcination temperature seems to be of particular interest (Table 1). The literature states that for conventional NMC442 or NMC333 materials, temperatures higher than 900 °C are necessary to obtain a material with good crystallinity and good performance [52,53]. In turn, for Ni-rich materials, it is characteristic for high temperatures above 800 °C to have a devastating effect on the properties of the cathode material, despite ensuring high crystallinity and a well-ordered structure. There is also no agreement in the literature regarding the method of calcination, i.e., the number of steps in which this process should be carried out to effectively ensure that favorable electrochemical parameters are obtained in the final cathode material (Table 1).

In this study, two series of Ni-rich layered oxides, i.e., LiNi_0.6_Mn_0.2_Co_0.2_O_2_ (NMC622) and LiNiO_2_ (LNO), were prepared at different temperatures using the two-step calcination procedure. A simulation of the calcination process of cathode material precursors was conducted in air over a wide range of temperatures using thermogravimetric analysis coupled with mass spectrometry (TGA-MS). Detailed structural and morphological characterization studies of the obtained samples were carried out using PXRD and SEM. The correlation of calcination temperature, structural properties and electrochemical performance of the studied Ni-rich materials was thoroughly investigated and discussed. The optimal conditions for NMC622 and LNO calcination are defined to ensure the most favorable electrochemical performance.

## 2. Materials and Methods

### 2.1. Preparation of LiNi_0.6_Mn_0.2_Co_0.2_O_2_ (NMC) and LiNiO_2_ (LNO) Cathode Materials

LiNi_0.6_Mn_0.2_Co_0.2_CO_3_ samples were prepared by impregnation of Ni_0.6_Mn_0.2_Co_0.2_CO_3_ with an aqueous solution of LiOH∙H_2_O (Chempur, Piekary Śląskie, Poland), as described in our previous paper [39]. Nickel-manganese-cobalt carbonate was prepared by co-precipitation method from an aqueous solution of NiSO_4_∙7H_2_O, MnSO_4_∙H_2_O and CoSO_4_∙7H_2_O (Chempur, Piekary Śląskie, Poland) (Ni^2+^/Mn^2+^/Co^2+^ = 6/2/2 molar ratio, the concentration was 2 mol L^−1^) and an aqueous solution of Na_2_CO_3_ (Chempur, Piekary Śląskie, Poland) (2 mol L^−1^) and NH_3_∙H_2_O (Chempur, Piekary Śląskie, Poland) (0.2 mol L^−1^). The solutions were mixed slowly in a reactor (capacity 2 L) filled with distilled water containing 1 wt% Polyvinylpyrrolidone (Sigma Aldrich, Saint Louis, MO, USA) The pH value was controlled in a range of 7.5–8.0; the temperature was set at 55 °C and stirring speed at 800 rpm during the precipitation process. After the precipitation, the slurry was aged, filtered, washed and dried at 120 °C for 18 h. The prepared Ni_0.6_Mn_0.2_Co_0.2_CO_3_ precursor (SSA = 97.5 m^2^ g^−1^) was impregnated with an aqueous solution of LiOH∙H_2_O (Chempur, Piekary Śląskie, Poland) at a molar ratio Li/TM = 1.05 (TM—transition metals). The excess of lithium hydroxide was added to compensate lithium evaporation during the process. The prepared samples (in the powder forms) were dried at 120 °C for 18 h and calcinated at 500 °C for 5 h and then at temperatures ranging from 750 to 950 °C for 15 h.

LiNiO_2_ samples were prepared by impregnation of NiO (Merck KGaA, Darmstadt, Germany) (SSA = 66.8 m^2^ g^−1^) with an aqueous solution of LiOH∙H_2_O (Li/TM = 1.05 molar ratio). The prepared samples (in powder form) were dried at 120 °C for 18 h and calcinated at 500 °C for 5 h and then at temperatures ranging from 650 to 850 °C for 15 h.

The list of the prepared samples and their corresponding Li/TM molar ratios is presented in Table 2.

### 2.2. Characterization Methods

A simulation of the calcination process of the cathode material precursors in air was conducted over a wide range of temperatures using thermogravimetric analysis coupled with mass spectrometry (TGA-MS). The measurements were carried out using the flow system of the STA449C thermobalance (NETZSCH, Selb, Germany) coupled with the QMS 403C Aëolos quadrupole mass spectrometer (NETZSCH, Selb, Germany). Samples of about 50 mg were subjected to heating at a constant rate of 5 °C min^−1^ under air flow (100 mL min^−1^) according to the following procedure: heating to 500 °C → isotherm at 500 °C (30 min) → heating to 750/800/850/900/950 °C → isotherm at 750/800/850/900/950 °C (90 min) → cooling to 30 °C for the precursors of the NMC samples. For the precursors of the LNO samples, the following procedure was applied: heating to 500 °C → isotherm at 500 °C (30 min) → heating to 650/700/750/800/850 °C → isotherm at 650/700/750/800/850 °C (90 min) → cooling to 30 °C. The temperature, weight losses and mass signals of selected gases (H_2_O, CO_2_) were continuously recorded during the measurements.

The specific surface area (SSA) of the prepared samples was determined using nitrogen physisorption method (ASAP 2020, Micromeritics Instrument Co., Norcross, GA, USA). Before the experiments, each sample was degassed under vacuum at 90 °C for 1 h and then at 300 °C for 4 h. The specific surface area was estimated by the Brunauer–Emmett–Teller method.

Laboratory powder X-ray diffraction patterns were recorded at room temperature on a Bruker D8 Advance diffractometer (Bruker Co., Billerica, MA, USA) equipped with a LYNXEYE position sensitive detector using Cu-Kα radiation (λ = 0.15418 nm). The data were collected in Bragg–Brentano (θ/θ) horizontal geometry (flat reflection mode) between 10° and 70° (2θ) in a continuous scan using 0.03° steps 960 s/step. The diffractometer incident beam path was equipped with a 2.5° Soller slit and a 1.14° fixed divergence slit, while the diffracted beam path was equipped with a programmable antiscatter slit (fixed at 2.20°), a Ni β-filter and a 2.5° Soller slit. The data were collected under the standard laboratory conditions (temperature and relative humidity).

The morphology of the prepared samples was characterized using a Field Emission Scanning Electron Microscope (FEI Nova NanoSEM 230, FEI Co., Hillsboro, OR, USA) equipped with energy-dispersive spectrometer (EDAX Genesis XM4, EDAX, Inc., Mahwah, NJ, USA). The prepared samples were dispersed in alcohol and then a drop was placed on the carbon stub. The stub was then heated in a vacuum oven for 30 min at 100 °C and subsequently cooled to the room temperature. To improve surface sensitivity and show more detailed features of the samples, SEM images were recorded in a beam deceleration mode at 5 kV.

The particle size distribution was determined using the dynamic light scattering method (DLS) using a Zetasizer Nano ZS (Malvern Panalytical, Malvern, UK). The measurements were carried out in the quartz cuvettes.

### 2.3. Electrochemical Evaluation

The electrochemical performances of the prepared cathode materials were measured using CR2032 coin-type half-cells (Lambda System, Warsaw, Poland) at room temperature assembled in an argon filled glovebox (< 1 ppm H_2_O, < 1 ppm O_2_). The electrodes were prepared by casting a slurry containing 0.5 g of the cathode powder, 0.05 g of carbon black (VULCAN^®^ XC72R, Cabot Co., Boston, MA, USA) and 1 g of 5 wt% solution of poly(vinylidene fluoride) (Sigma Aldrich, Saint Louis, MO, USA) in ca. 500 µL of 1-Methyl-2-pyrrolidinone (Sigma Aldrich, Saint Louis, MO, USA) onto an aluminum foil (20 μm thick, Hohsen Corp., Tokyo, Japan) and were pre-dried at 80 °C overnight and then vacuum dried for 24 h (120 °C, < 1 mbar). After drying, the electrodes were punched into discs (12.8 mm in diameter) and pressed (6 t cm^−2^). Electrochemical half-cells were assembled with the cathodes as synthesized, metallic lithium foil (99.9%, Sigma Aldrich, Saint Louis, United States of America) as an anode, and 1.0 M LiPF_6_ in a EC:DMC (1:1) solution (99.9%, max. 20 ppm H_2_O, Solvionic, Toulouse, France) as an electrolyte. Galvanostatic cycling was performed between 3 to 4.4 V vs. Li^+^/Li and at C-rate between 0.05C to 5C using a BioLogic multi-channel battery cycler (Biologic, Seyssinet-Pariset, France) at room temperature.

## 3. Results and Discussion

### 3.1. Thermal Decomposition of the Prepared Precursors of Cathode Materials

**Figure 1:**
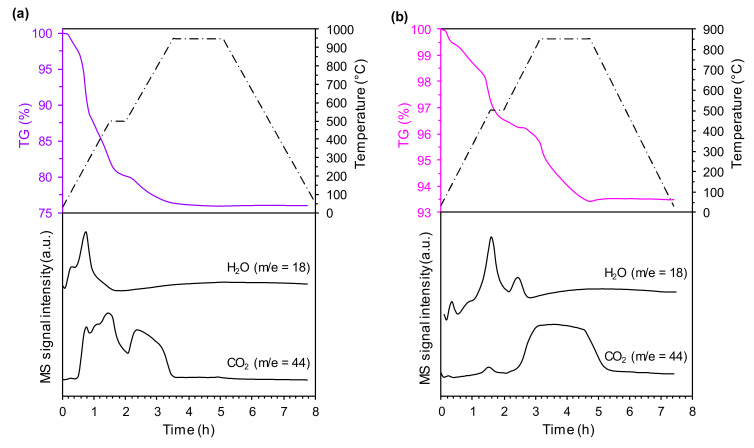
TGA-MS curves of (**a**) Ni_0.6_Mn_0.2_Co_0.2_CO_3_ and LiOH∙H_2_O and (**b**) NiO and LiOH∙H_2_O mixtures.

Figure 1 shows the TGA-MS curves of the precursors of LiNi_0.6_Mn_0.2_Co_0.2_O_2_ (Ni_0.6_Mn_0.2_Co_0.2_CO_3_ and LiOH∙H_2_O mixture) and LiNiO_2_ (NiO and LiOH∙H_2_O mixture) recorded at selected heat-treatment (calcination) conditions, i.e., the first step at 500 °C and the second step at 950 °C for the NMC precursor and the first step at 500 °C and the second step at 850 °C for the LNO precursor. The analogous courses of TGA-MS curves were recorded for both precursor mixtures regardless of the heat-treatment (calcination) conditions, and thus they are not shown. For the precursor of the NMC samples (Figure 1a) the mechanism for the thermal decomposition is as follows:

(i) lithium hydroxide monohydrate dehydrates to lithium hydroxide and water at 100 °C. The confirmation is a mass signal of water (m/e = 18).
(ii) nickel–manganese–cobalt hydroxy carbonate decomposes from 150 to 500 °C, along with the decomposition of lithium hydroxide. These thermal decomposition reactions are associated with releasing water (m/e = 18) and carbon dioxide (m/e = 44).
(iii) nickel–manganese–cobalt carbonate decomposes from 500 to 900 °C, along with decomposition of lithium carbonate (formed in the reaction between lithium hydroxide and carbon dioxide (contained in air) during the drying step). The confirmation is a mass signal of carbon dioxide (m/e = 44).

For the precursor of the LNO samples (Figure 1b), the mechanism for the thermal decomposition is as follows:

(i) lithium hydroxide monohydrate dehydrates at 100 °C and water (m/e = 18) is released.
(ii) lithium hydroxide decomposes to lithium oxide and water at 500 °C. The confirmation is a mass signal of water (m/e = 18).
(iii) lithium hydroxy carbonate and lithium carbonate (formed in the reaction between lithium hydroxide and carbon dioxide (contained in air) during the drying step) decompose from 500 to 850 °C. These thermal decomposition reactions are related to the release of water (m/e = 18) and carbon dioxide (m/e = 44).

Table 3 presents the weight losses recorded for the precursors of the NMC and LNO samples at different heat-treatment (calcination) conditions. For the precursors of the NMC samples, the weight losses were comparable, regardless of the heat-treatment (calcination) conditions, indicating that the required thermal decomposition reactions occur at temperatures below 750 °C. Comparing the weight losses of the precursors of the LNO samples, some discrepancies can be observed. Increasing the temperature of the second step of the calcination process induced higher weight loss, which is associated with the thermal decomposition of lithium hydroxy carbonate/lithium carbonate requiring temperatures above 750 °C. Note that the weight losses recorded for the precursors of the NMC samples were higher than those for the precursors of the LNO samples. This is related to the form in which the transition metals in the precursor of the NMC samples occur. It is necessary to transform nickel–manganese–cobalt carbonate into the oxide form before it reacts with lithium compounds, forming lithiated nickel–manganese–cobalt oxide.

### 3.2. Physicochemical Properties

Table 2 depicts the chemical composition of the prepared samples. The results revealed no lithium deficiencies for the NMC and LNO samples regardless of the calcination temperature (Li/TM molar ratios are greater than unity). However, the higher the calcination temperature for the NMC and LNO cathodes, the higher the reduction of lithium content observed due to its evaporation. Note that a higher decrease in Li/TM molar ratio was recorded in the NMC samples, which is associated with their higher calcination temperatures compared to the LNO samples.

For both the NMC and LNO cathodes, the values of specific surface area (SSA) decrease with increasing calcination temperature (Table 4). This is particularly prominent for the NMC samples, in which increasing the calcination temperature from 750 to 950 °C led to an approximately 7-fold decrease in the values of SSA, while for the LNO, increasing calcination temperature from 650 to 850 °C led to an approximately 3-fold decrease in the values of SSA. This is related to the excessive sintering process of the NMC samples’ particles during the calcination process at high temperatures. However, the values of SSA of the NMC samples are larger than those for the LNO samples. This is presumably associated with the larger quantities of gaseous compounds (water vapor and carbon dioxide) released during the calcination stage compared to the precursors of the LNO samples, which act as pore-forming agents.

Comparing the values of tap density, discrepancies were observed. As shown in Table 4, the increase of calcination temperature for both the NMC and LNO samples led to an increase in tap density, which is related to the reduction of porosity by excessive sintering processes.

### 3.3. Structural Properties

**Figure 2:**
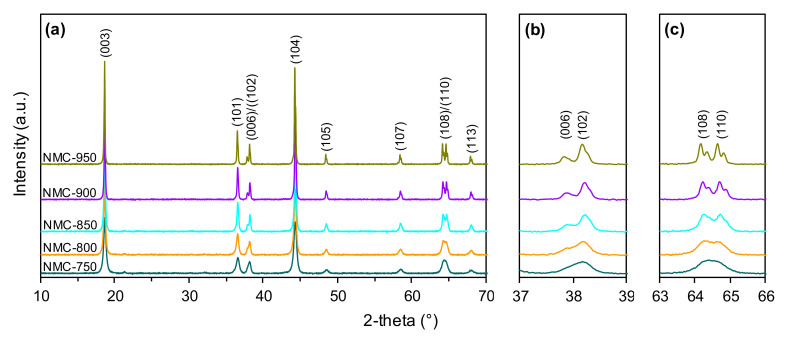
PXRD patterns (**a**) and magnified view of (006)/(102) (**b**) and (108)/(110) (**c**) diffraction peaks of the NMC samples prepared at different calcination temperatures.

Figure 2a presents the PXRD patterns of the NMC samples calcined at different temperatures ranging from 750 to 950 °C. For all the studied samples, the main diffraction peaks can be indexed to hexagonal α-NaFeO_2_ type structures with an R3m space group [54]. However, when the calcination temperature increases, the reflections become sharper (smaller full width at half maximum) and more distinct. This proves the increase in the crystallinity of the studied NMC cathode materials and indicates that a more ordered hexagonal layered structure was obtained. This is also confirmed by the characteristic splitting of (006)/(102) and (108)/(110) peaks at about 38° (Figure 2b) and 65° (Figure 2c), respectively, which becomes more pronounced as the calcination temperature gradually increases [48]. An additional peak of a very low intensity is visible at 21° and can be ascribed to the presence of Li_2_CO_3_. However, this impurity disappears for the samples calcined at 900 and 950 °C.

**Figure 3:**
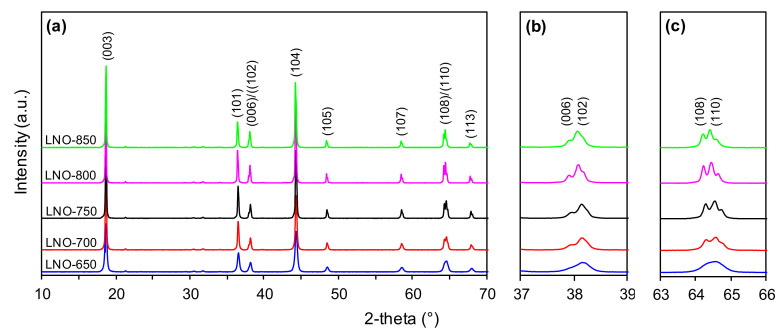
PXRD patterns (**a**) and magnified view of (006)/(102) (**b**) and (108)/(110) (**c**) diffraction peaks of the LNO samples prepared at different calcination temperatures.

Analogous structural relationships were observed on the PXRD profiles of the LNO cathode materials calcined in the temperature range of 650–850 °C (Figure 3a). Sharper reflections and much better splitting of (006)/(102) and (108)/(110) peaks (Figure 3b,c, respectively) were observed with increasing calcination temperature. Additionally, additional impurity (Li_2_CO_3_) peaks are visible in the range of 21–32°, but unlike the NMC samples (Figure 2a) they did not disappear with increased calcination temperature.

The main structural parameters obtained from the Rietveld refinement of the PXRD data for all of the studied samples are summarized in Table 5. They clearly confirm that a higher calcination temperature of the cathode materials favors the formation of the well-ordered layered structure necessary for the efficient intercalation/deintercalation of lithium ions during the battery charge/discharge cycles. This is evidenced by the values of the c/a ratio, which for all the studied materials are higher than 4.899 [55]. However, there are some differences in the lattice parameters of the NMC and LNO cathode materials. For the former, the calculated a and c parameter values, as well as the cell volume (V), decrease with increasing calcination temperature, but only to a temperature of 900 °C, beyond which the values of all of these parameters increase. For the latter, a constant increase in the values of the a, c and V parameters was observed with increasing calcination temperature, which can be ascribed to the enhanced crystallinity of the studied LNO cathode materials. The average crystallite sizes calculated based on the Scherrer equation (k = 0.89) were in the range of 17–94 and 28–124 nm for the NMC and LNO samples, respectively. Moreover, the I_003_/I_104_ intensity ratio (R parameter), which is inversely proportional to the degree of Ni^2+^/Li^+^ cation mixing [48,56], increases with increasing calcination temperature, reaching its highest value for the NMC-900 and LNO-700 samples, and then decreases. This means that the samples calcined at 900 °C for the NMC and 700 °C for the LNO powders, respectively, showed the lowest cation mixing, i.e., the lowest amount of Ni^2+^ ions located at sites normally occupied by Li^+^ ions, in the interlayer spaces. The observed decrease in R values for the samples calcined at higher temperatures, i.e., 950 °C for the NMC and 750–850°C for the LNO materials, may indicate that the temperature increase may lead to an intensified release of lithium and the related formation of non-stoichiometric Li_1-x_Ni_0.6_Co_0.2_Mn_0.2_O_2_ and Li_1-x_NiO_2_ compounds. This is in a good agreement with the results of the analysis of the chemical composition of the obtained materials (Table 2), indicating a decrease in lithium content with the increase in calcination temperature of the tested cathode materials. A temperature of 900 °C for the LiNi_0.6_Co_0.2_Mn_0.2_O_2_ and 700 °C for the LiNiO_2_ may be considered optimal for obtaining a cathode material with promising electrochemical properties. However, as shown by the PXRD results, it is important to strike a balance between structural crystallinity and cation disorder. A high degree of crystallinity accompanied by strong cation mixing, as is the case for the LNO materials calcined at temperatures higher than 700 °C, may be detrimental to the final electrochemical performance of the studied materials.

### 3.4. Morphological Properties

**Figure 4:**
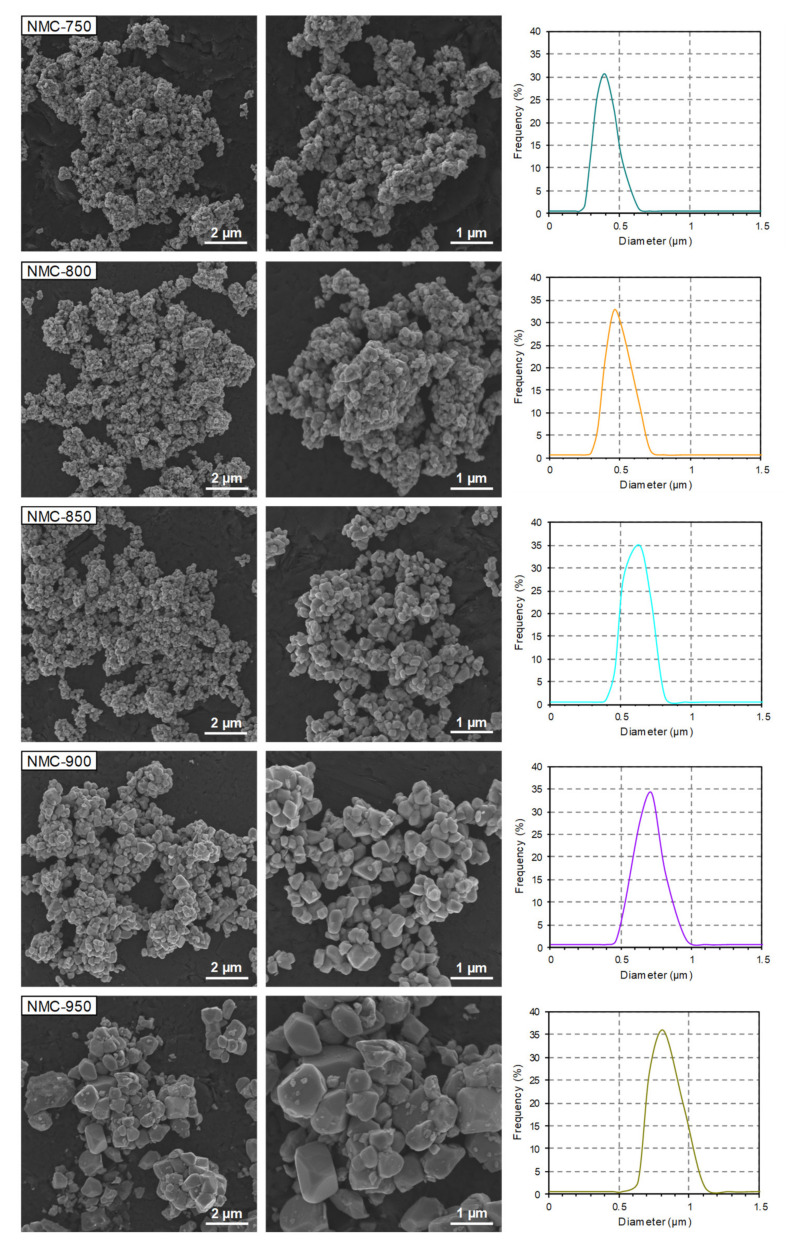
SEM images and particle size distribution curves of the NMC samples prepared at different calcination temperatures.

Figure 4 depicts the SEM images and particle size distribution curves of the NMC samples. All the samples are composed of irregular agglomerates/aggregates, and estimated average particle sizes are between 0.1 and 1.2 μm. With increasing calcination temperature, prominent particle growth was observed, and the samples showed explicit grain boundaries between particles (especially between 900 and 950 °C). The particle size was up to 0.7 μm for the NMC-900 sample, and this increased to 1.2 μm for the NMC-950 sample. These observations are consistent with the particle size distribution curves (Figure 4). It can be observed that with increasing calcination temperature, the curves shift towards higher particle diameter values, along with increasing average particle sizes and average crystallite sizes (Table 4 and Table 5).

**Figure 5:**
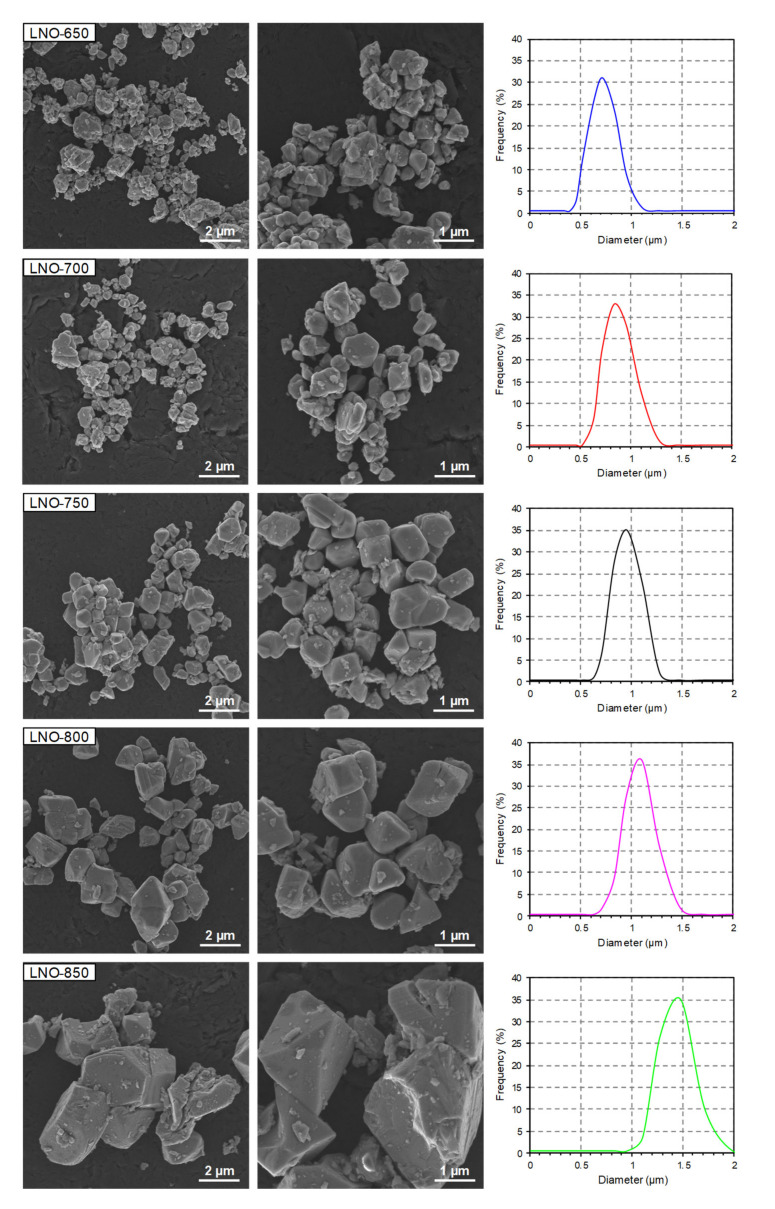
SEM images and particle size distribution curves of the LNO samples prepared at different calcination temperatures.

Analogous observations were made for the LNO samples (Figure 5). All of the samples were composed of well crystallized and irregular particles with obvious agglomeration/aggregation. The estimated average particle sizes were between 0.5 and 2 μm. With increasing calcination temperature, the particles exhibit a growing trend with respect to size (especially between 800 and 850 °C), which is consistent with the particle size distribution curves (Figure 5) and the estimated average particle size and average crystallite size (Table 4 and Table 5). Although the NMC and LNO cathode materials possess the same morphology, the NMC samples are composed of smaller particles compared to the LNO samples (despite higher calcination temperatures being used in the NMC samples). This presumably indicates that cathode materials with high nickel content have a strong tendency towards crystallite/particle growth with increasing calcination temperature.

### 3.5. Electrochemical Performance

To determine the influence of the calcination temperature on the electrochemical performance of the studied NMC and LNO materials, the prepared electrodes were subjected to galvanostatic cycling with potential limitations at the voltage range of 2.8–4.4 V vs. Li^+^/Li and with different C-rates. It can be clearly seen (Table 6) that the highest initial capacity value is exhibited by the NMC-900 cathode powder calcined at a temperature of 900 °C. The initial charge and discharge capacities at 0.05C for this material were 245 and 186 mAh g^−1^, respectively. The materials prepared at lower temperatures (the NMC-750, NMC-800, NMC-850 samples) showed initial charge/discharge capacities ca. 20% lower than those for the NMC-900 cathode powder, and as a result, they also exhibited lower coulombic efficiency. It is worth emphasizing that the material calcined at the highest temperature of 950 °C (the NMC-950 sample) had an initial charge/discharge capacity that was even 35% lower than that of the NMC-900 material. This is related to the increased lithium loss, high degree of Ni^2+^/Li^+^ cation mixing (Table 5) and increased average crystallite and particle sizes (Table 4 and Table 5), which may imply an extension of the Li^+^ ion diffusion pathway. The series of LNO cathode materials exhibited worse electrochemical performance than the studied NMC cathode powders. In general, when the calcination temperature increases, the capacity for the LNO samples gradually decreases. The highest initial charge/discharge capacities were found for the materials calcined at lower temperatures, i.e., 650 and 700 °C (the LNO-650 and LNO-700 samples). For the samples calcined at higher temperatures in the range of 750–850 °C (the LNO-750, LNO-800 and LNO-850 samples), the values of the initial charge/discharge capacity were ca. 10–35% lower than those for the samples calcined at lower temperatures.

**Figure 6:**
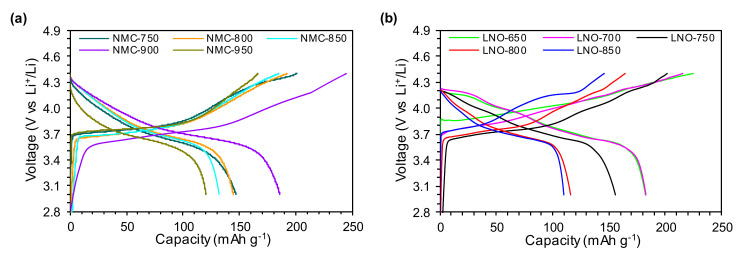
Initial charge/discharge curves at 0.05C between 2.8 and 4.4 V for the (**a**) NMC and (**b**) LNO samples prepared at different calcination temperatures.

The initial charge/discharge curves for the studied materials between 2.8–4.4 V at 0.05C are shown in Figure 6. For the series of NMC cathode materials calcined at temperatures of 750, 800, 850 and 950 °C (the NMC-750, NMC-800, NMC-850, NMC-950 samples), the shapes of the charge/discharge curves (Figure 6a) are similar, regardless of the calcination temperature. A potential plateau can be observed in the charge curves at about 3.7 V vs. Li^+^/Li. For the NMC-900 sample alone, this plateau is visible at about 3.5 V. When the voltage exceeded 4.1 V, a characteristic bend was present in all of the studied samples. In the case of the LNO samples (Figure 6b), the initial charge curves are much more complicated than for the NMC materials. The potential plateau is visible at 3.85 V for the LNO-650, 3.7 V for the LNO-700 and LNO-850, and 3.6 V for the LNO-750 and LNO-800 samples. The most stable course of the charge curve is observed for the materials calcined at lower temperatures, i.e., 650 and 700 °C (the LNO-650, LNO-700 samples). Many bends are present in the charge curves of the materials calcined at higher temperatures in the range of 750–850 °C (the LNO-750, LNO-800, LNO-850 samples) when the voltage is increased beyond 3.7 V, which may be caused by structural damage occurring as a result of charging to the high upper cutoff voltage [26].

**Figure 7:**
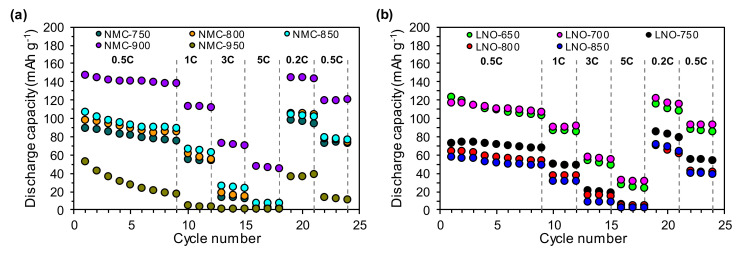
Rate capability performance of the (**a**) NMC and (**b**) LNO samples prepared at different calcination temperatures.

Figure 7 presents the rate capability of the studied cathode materials. Among all of the studied NMC samples (Figure 7a), the NMC-900 sample showed the highest capacity, with 148 mAh g^−1^ at 0.5C. This material is also characterized by good cyclic stability; over 10 cycles at 0.5C, it retained 95% of its capacity. When increasing the discharge rate to 1C, 3C or 5C, the capacity of this cathode powder was reduced by about 35% to 40%. The materials calcined at lower temperatures in the range of 750–850 °C (the NMC-750, NMC-800, NMC-850 samples) were characterized by lower capacities than the NMC-900 material. However, these three samples exhibited similar characteristics both in terms of capacity (in the range of 90–110 mAh g^−1^) and cyclic stability (capacity retention of 80% after 10 cycles at 0.5C). The sample calcined at the highest temperature of 950 °C (the NMC-950 sample) had significantly different and highly unfavorable properties compared to the other NMC materials. It had a very low capacity of 53 mAh g^−1^ in the first cycle at 0.5C. After 10 cycles at 0.5C, it had lost almost 70% of its capacity, and when the discharge rate was increased to 1C or more, it almost completely lost its capacity.

Overall, the series of LNO samples (Figure 7b) exhibited worse electrochemical performance than the NMC cathode powders (Figure 7a). However, among the LNO samples themselves, significant differences were visible in their properties depending on the temperature of their calcination. These results indicated that the samples calcined at lower temperatures of 650–700 °C (the LNO-650 and LNO-700 samples) achieved comparable results to the highest capacity of about 120 mAh g^−1^ at 0.5C. Only the capacity retention was slightly different—85% for the LNO-650 and 92% for the LNO-700 sample. As the calcination temperature increased, the capacity of the LNO materials gradually decreased, i.e., the capacity for the LNO-750, LNO-800 and LNO-850 samples was approximately 25–35% lower than for the LNO-650 and LNO-700 materials. The same tendency was observed in the case of capacity retention—after 10 cycles at 0.5C it was 92%, 83% and 85% for the LNO-750, LNO-800 and LNO-850 samples, respectively.

The development of a new generation of lithium-ion cells is possible mainly through improvements in the performance parameters of cathode materials. The most important requirements for cathode materials are associated primarily with their high capacity, good ion-electron conductivity, chemical stability and reversibility of a lithium ion intercalation/deintercalation process. Cathode materials based on mixed transition metal oxides of the LiNi_x_Mn_y_Co_z_O_2_ type are widely recognized by both scientists and the battery industry for two main reasons, i.e., their layered structure, which provides favorable two-dimensional diffusion channels for lithium ions during battery charging/discharging cycles [57], and good electrochemical properties resulting from the synergistic action of all their components: nickel, manganese and cobalt [58]. Nickel is responsible for the high capacity of the cathode. Therefore, there is a tendency to increase the nickel content in a cathode powder at the expense of other components (especially cobalt, which is a toxic and expensive raw material), which is entirely justified. Hence, the growing interest in LiNiO_2_ (LNO) material as a future generation of cathode materials for Li-ion batteries. However, the high nickel content also has some negative implications—including the performance degradation of cathodes, and their status as a safety hazard—that are directly related to the occurrence of serious problems that are characteristic of Ni-rich materials [19]. One of the most important is the phenomenon of Ni^2+^/Li^+^ cation mixing, which involves the migration of low-valence metal ions into interlayer spaces normally occupied by Li ions. Nickel ions are easily translocated from 3b sites to 3a sites due to the significant similarity of Ni^2+^ (0.69 Å) to Li^+^ (0.76 Å) ions. According to literature reports [19,59,60,61], this phenomenon causes a serious decline in capacity and reduced mobility of Li^+^ ions. It is also postulated that there is then a change in the crystallographic structure from layered by spinel into a NiO-like rock salt phase [19,26,62,63]. The stability of the layered structure is, in turn, a crucial factor necessary for effective and reversible intercalation/deintercalation of lithium ions during battery working cycles.

In view of the foregoing, there is no doubt that the composition of the cathode material plays a key role in its properties, but also in the performance and stability of the entire Li-ion battery. In the present research, we synthesized, characterized and compared the properties of two types of Ni-rich cathode powders: NMC622 and LNO. Surprisingly, the higher nickel content in the LNO materials in comparison to the NMC materials proved not to be beneficial—lower capacity and stability were observed in the LNO powders than in the NMC cathode materials. The following reasons can be proposed for this deterioration in electrochemical performance:

(i) the charging voltage was too high, causing an irreversible phase transformation. The literature indicates that a course of charging process related to a successive phase transformation occurring in the sequence hexagonal (H1) → monoclinic (M) → hexagonal (H2) → hexagonal (H3) [19,26,62,63] is also important in the phenomenon of capacity fading. Depending on the composition (nickel content particularly) of the cathode material, the potential for the last irreversible H2 → H3 transformation decreases. For the NMC622 material, this potential is higher than 4.6 V vs. Li/Li^+^. However, for materials with higher nickel content (x ≥ 0.8), it is 4.2–4.3 V [19,61] and can be as low as 4.0 V vs. Li/Li^+^ for LNO materials [19,26]. This suggests that the charging of the studied materials during galvanostatic testing at high voltages of 4.4 V could also have an impact on the decrease of the capacity and stability of the LNO samples and lead to gradual cell degradation.
(ii) the presence of impurities in the form of Li_2_CO_3_, which may significantly reduce the amount of working lithium ions during the charge/discharge process. Residual lithium compounds are inevitably present in the Ni-rich layered oxides, and their amount increases with the Ni content [19].
(iii) Ni^2+^/Li^+^ cation mixing is more intense when the nickel content in the cathode powder is increased. The capacity and Li^+^ ion mobility decrease, and the crystal structure is transformed from a layered over spinel to a NiO-like rocksalt phase [19,60,61,62,64].
(iv) changes in the texture and microstructure of the materials, i.e., a deterioration of their specific surface area, as well as greater tendency for particles to aggregate/agglomerate, are highly adverse for the process of lithium ion diffusion, and are visible with increasing nickel content.

All the above-mentioned phenomena are closely related to the applied calcination procedure for cathode powder precursors. Heat treatment (calcination) is one of the most important steps in the preparation of these materials and is capable of determining their crucial properties. The correlation of calcination temperature, microstructure and the electrochemical performance of cathode materials has been proved in literature reports with respect to different calcination conditions in the heat-treatment of NMC materials (Table 1) [45,46,47,48,49]. In general, an increase in the calcination temperature of high-nickel content materials seems to be beneficial from the point of view of creating a highly ordered layered structure. This was also indicated by the results of the present PXRD studies (Table 5, Figure 2 and Figure 3). However, this may also be accompanied by the intensification of the cation mixing effect, causing substantial performance degradation at the same time. According to Du et. al. [65], the increasing lattice parameters can enhance lithium-ion migration in the crystal lattice, improving the electrochemical performance of the cathodes. Despite increased values of the c parameter being observed for the selected cathodes (with increasing calcination temperature), this was not reflected in the improvement of electrochemical performances. This is associated with the strong tendency for Ni^2+^/Li^+^ cation mixing with increasing calcination temperature, and this effect may be predominant over the improved structural characteristics of the cathodes. The favorable capacity and stable performance are also strictly associated with advantageous surface properties, particles of an appropriate size, and the low tendency for agglomeration/aggregation. As can be seen for the tested NMC and LNO materials, all of these characteristics are determined by the temperature of the heat-treatment of the precursors during preparation. It is, therefore, important to find the right temperature conditions in order to achieve optimal structural and electrochemical properties. The presented research indicates that with increasing nickel content, the optimal calcination temperature shifts to lower temperatures, and this was 900 °C for the NMC622 and 700 °C for the LNO. This demonstrates that the optimal calcination temperature is strongly dependent on the chemical compositions of the cathode materials itself. Hence, the perspective of the development of the efficient Ni-rich materials requires the optimization of the production procedure in terms of heat-treatment (calcination) conditions for each cathode material separately.

## 4. Conclusions

In summary, a series of Ni-rich (NMC622 and LNO) layered oxides were successfully prepared at different calcination temperatures using the two-step calcination procedure. The optimal calcination temperatures for the NMC622 and LNO samples were determined to be 900 °C and 700 °C, respectively. The prepared NMC-900 and LNO-700 samples possessed a highly ordered layered structure, reduced Ni^2+^/Li^+^ cation mixing, and good electrochemical performance. The results highlight the importance of a selection of calcination temperature for the preparation of Ni-rich cathode materials for Li-ion batteries. This demonstrates that the optimal calcination temperature is dependent on the chemical compositions of the cathode materials themselves. With increasing nickel content, the optimal calcination temperature shifts to lower temperatures, and this has to be taken into account when preparing Ni-rich cathode materials.

## Figures and Tables

**Table 1:** Electrochemical performances of the selected cathode materials prepared at different calcination temperatures [45,46,47,48,49].

Cathode Material	Heat-Treatment (Calcination) Conditions		Electrochemical Performance			Ref.
			Initial Discharge Capacity (mAh g^−1^)	Capacity Retention (%)	Voltage Range (V vs. Li/Li^+^)	
LiNi_0.5_Co_0.2_Mn_0.3_O_2_	two-step	500 °C (4 h)/800–900 °C (12 h)	150–163 (0.1C)	>99 (30 cycles, 0.2C)	2.5–4.3	[46]
LiNi_0.6_Co_0.2_Mn_0.2_O_2_	two-step	650 °C (6 h)/750–850 °C (10–12 h)	130–165 (1C)	>99 (100 cycles, 1C)	3.0–4.3	[47]
Li_1.2_Mn_0.56_Ni_0.16_Co_0.08_O_2_	one-step	750–950 °C (20 h)	255–269 (0.1C)	84–102 (100 cycles, 0.1C)	2.0–4.8	[48]
LiNi_0.76_Mn_0.14_Co_0.10_O_2_	two-step	500 °C (10 h)/725–900 °C (20 h)	200–215 (0.1C)	42–80 (200 cycles, 0.3C)	2.7–4.5	[45]
LiNi_0.5_Mn_0.3_Co_0.2_O_2_	one-step	900 °C (15 h)	161 (0.1C)	59 (30 cycles, 0.1C)	2.8–4.3	[49]
LiNi_0.5_Mn_0.3_Co_0.2_O_2_	two-step	450 °C (6 h)/900 °C (15 h)	171 (0.1C)	69 (30 cycles, 0.1C)	2.8–4.3	[49]
LiNi_0.5_Mn_0.3_Co_0.2_O_2_	two-step	750 °C (6 h)/900 °C (15 h)	165 (0.1C)	63 (30 cycles, 0.1C)	2.8–4.3	[49]
LiNi_0.5_Mn_0.3_Co_0.2_O_2_	three-step	450 °C (3 h)/560 °C (3 h)/900 °C (15 h)	179 (0.1C)	85 (30 cycles, 0.1C)	2.8–4.3	[49]

**Table 2:** List of the prepared samples.

Samples	Calcination Conditions		Li/TM Molar Ratio ^1^
	1st Step	2nd Step	
NMC-750	500 °C, 5 h	750 °C, 15 h	1.024
NMC-800	500 °C, 5 h	800 °C, 15 h	1.019
NMC-850	500 °C, 5 h	850 °C, 15 h	1.012
NMC-900	500 °C, 5 h	900 °C, 15 h	1.007
NMC-950	500 °C, 5 h	950 °C, 15 h	1.002
LNO-650	500 °C, 5 h	650 °C, 15 h	1.048
LNO-700	500 °C, 5 h	700 °C, 15 h	1.041
LNO-750	500 °C, 5 h	750 °C, 15 h	1.033
LNO-800	500 °C, 5 h	800 °C, 15 h	1.026
LNO-850	500 °C, 5 h	850 °C, 15 h	1.018

^1^ Li/TM molar ratio determined by inductively coupled plasma optical emission spectroscopy (ICP-OES).

**Table 3:** Weight losses derived from TGA-MS curves for Ni_0.6_Mn_0.2_Co_0.2_O_2_ and LiOH∙H_2_O (NMC) and NiO and LiOH∙H_2_O (LNO) mixtures at different temperature ranges.

Samples	Temperature Range (°C)	Weight Loss (%)
NMC-750	30–750	24.1
NMC-800	30–800	24.1
NMC-850	30–850	24.2
NMC-900	30–900	24.4
NMC-950	30–950	24.5
LNO-650	30–650	3.8
LNO-700	30–700	4.4
LNO-750	30–750	4.9
LNO-800	30–800	5.1
LNO-850	30–850	5.5

**Table 4:** Physicochemical properties of the prepared NMC and LNO samples.

Samples	SSA ^1^ (m^2^ g^−1^)	D_av_^2^ (µm)	Tap Density (cm^3^ g^−1^)
NMC-750	8.9	0.4	1.31
NMC-800	8.1	0.5	1.35
NMC-850	5.5	0.6	1.39
NMC-900	1.5	0.7	1.51
NMC-950	1.2	0.9	1.78
LNO-650	1.6	0.7	2.16
LNO-700	1.2	0.8	2.17
LNO-750	0.9	1.0	2.20
LNO-800	0.7	1.1	2.22
LNO-850	0.6	1.5	2.29

^1^ specific surface area (SSA) estimated based on the Brunauer–Emmett–Teller (BET) method; ^2^ average particle diameter (D_av_) estimated by the dynamic light scattering (DLS) method.

**Table 5:** Rietveld refinement structural data of the NMC and LNO samples prepared at different calcination temperatures.

Samples	a (Å)	c (Å)	V (Å^3^)	c/a	R ^1^	d_av_^2^ (nm)
NMC-750	2.881	14.237	102.34	4.942	1.09	17
NMC-800	2.879	14.235	102.18	4.944	1.13	25
NMC-850	2.878	14.235	102.09	4.946	1.19	37
NMC-900	2.879	14.243	102.20	4.947	1.21	63
NMC-950	2.880	14.248	102.33	4.947	1.03	94
LNO-650	2.880	14.205	102.07	4.932	1.16	28
LNO-700	2.882	14.208	102.19	4.923	1.18	56
LNO-750	2.883	14.207	102.28	4.928	1.12	75
LNO-800	2.886	14.212	102.54	4.924	1.08	96
LNO-850	2.889	14.218	102.73	4.921	1.06	124

^1^ R = I_003_/I_104._; ^2^ d_av_–average crystallite size.

**Table 6:** Initial charge/discharge capacity at 0.05C for the NMC and LNO samples prepared at different calcination temperatures.

Samples	Charge Capacity (mAh g^−1^)	Discharge Capacity (mAh g^−1^)	Coulombic Efficiency (%)
NMC-750	201	147	73
NMC-800	192	145	76
NMC-850	185	132	71
NMC-900	245	186	76
NMC-950	166	120	72
LNO-650	225	182	81
LNO-700	215	183	85
LNO-750	202	156	77
LNO-800	164	116	71
LNO-850	146	110	75
